# Stable elastic nail application with poller K-wire for Irreducible distal radius metaphyseal-diaphyseal Junction fractures in preadolescents: a new operative technique

**DOI:** 10.1186/s12891-024-07358-5

**Published:** 2024-03-21

**Authors:** Levent Horoz, Mehmet Fevzi Cakmak, Cihan Kircil

**Affiliations:** Faculty of Medicine Orthopedics and Traumatology Clinic, Kırşehir Ahi Evran University, Kirsehir, Turkey

**Keywords:** Elastic stable intramedullary nailing, Diaphyseal metaphyseal junction, Pediatric, Distal radius fracture

## Abstract

**Background:**

Surgical treatment of irreducible distal radius diaphyseal- metaphyseal junction fractures involves difficulties as the fracture remains too proximal for K-wire fixation and too distal for the elastic stable intramedullary nail. Our study aims to present the clinical results of applying an elastic stable intramedullary nail with a poller K-wire to achieve both reduction and stable fixation.

**Patients and methods:**

A retrospective analysis was performed on 26 patients who underwent ESIN with a poller K-wire for distal radius diaphyseal-metaphyseal region fracture. Reduction parameters such as residual angulation and alignment were evaluated on postoperative follow-up radiographs. Changes in angular and alignment parameters on follow-up radiographs were recorded. Wrist and forearm functions were evaluated at the last follow-up.

**Result:**

There were 17 male and nine female patients with an average age of 10.9. The residual angulation in coronal and sagittal planes on immediate postoperative radiographs was 4.0 ± 1.62° and 3.0 ± 1.26°, respectively. The mean translation rate on immediate postoperative radiographs was 6.0 ± 1.98% and 5.0 ± 2.02% in the coronal and sagittal planes, respectively. No change was observed in translation rates in the last follow-ups. The mean angulation in the coronal and sagittal planes measured on 6th-week radiographs was 4.0 ± 1.72°and 3.0 ± 1.16°, respectively. No significant difference was observed in angular changes in the sagittal and coronal planes at the last follow-up (*p* > 0.05). No tendon injury or neurovascular injury was observed in any of the patients.

**Conclusion:**

In the surgical treatment of pediatric DRDMJ fractures, applying ESIN with poller K-wire is an effective, safe, and novel method for achieving reduction and stable fixation.

## Introduction

Distal radius fractures are the most common fractures in the pediatric population [1]. It accounts for 35% of all pediatric fractures [2]. Although pediatric distal radius fractures can be successfully treated conservatively, reduction losses of 21–39% can be observed in the treatment with a plaster cast [3].

Reduction losses are frequently observed in distal radius diaphyseal metaphyseal junctional (DRDMJ) fractures, primarily due to the limited contact surface of the fracture [4]. There has yet to be a consensus regarding the treatment protocol for DRDMJ fractures [5]. In DRDMJ fractures, surgery is recommended in cases where satisfactory alignment cannot be achieved with closed reduction and cast immobilization, or there is reduction loss in clinical follow-ups [4, 6, 7].

In the surgical treatment of pediatric distal radius fractures, despite achieving successful outcomes with closed reduction and K-wire fixation, these techniques prove inadequate for fractures occurring at the diaphysis-metaphysis junction (DMJ). Reduction losses can be observed [8]. Due to the limited length of the distal segment in pediatric distal radius fractures, retrograde elastic stable intramedullary nailing (ESIN) application leads to displacement at the fracture site [8]. Lieber et al. [9] emphasized that DRDM fractures are too proximal for fixation with a K-wire and too distal for fixation with an ESIN. Reduction losses are observed after inadequate fixation of DMJ fractures [10]. The narrowness of the DRDM region makes K-wire applications more complicated than the metaphyseal region. As a result of the application of the K-wire’s higher insertion angle and applying the K-wire closer to the fracture line, the fixation strength decreases. In diaphyseal radius fractures, the deforming effect of ESIN decreases depending on the application since it bends very distal to the fracture line, and ESIN is already directed towards the intramedullary area. In DRDMJ fractures, on the other hand, the stress caused by the bending of the ESIN is close to the fracture line, leading to angulation. When the dorsal entry point is preferred for the ESIN in DRDMJ fractures, the tensile forces due to bending of the ESIN cause flexion of the fracture line in the sagittal plane. When a more radial entry point is preferred, it causes coronal plane deformities. Many intramedullary surgical techniques have been described due to inadequate K-wire fixation and displacement due to ESIN applications [4, 9, 11]. It can lead to severe but rare complications such as growth plate disorder and iatrogenic fracture associated with these surgical techniques. Considering skeletal maturity, open reduction and internal fixation (OR-IF) are becoming popular in current practice in older pediatric cases [12]. In our clinical practice, although we prefer OR-IF in patients over 14 years of age or who have completed skeletal maturity, we use more minimally invasive intramedullary fixation methods in cases that have not completed skeletal maturity.

In this article, the focus is on the implementation of ESIN’s poller K-wire technique to prevent fracture displacement and achieve stable fixation during clinical follow-ups. Our study aims to report this novel technique’s effectiveness and clinical outcomes, which we have defined for DRDMJ fractures.

## Patients and methods

### Study Population

**Fig. 1:**
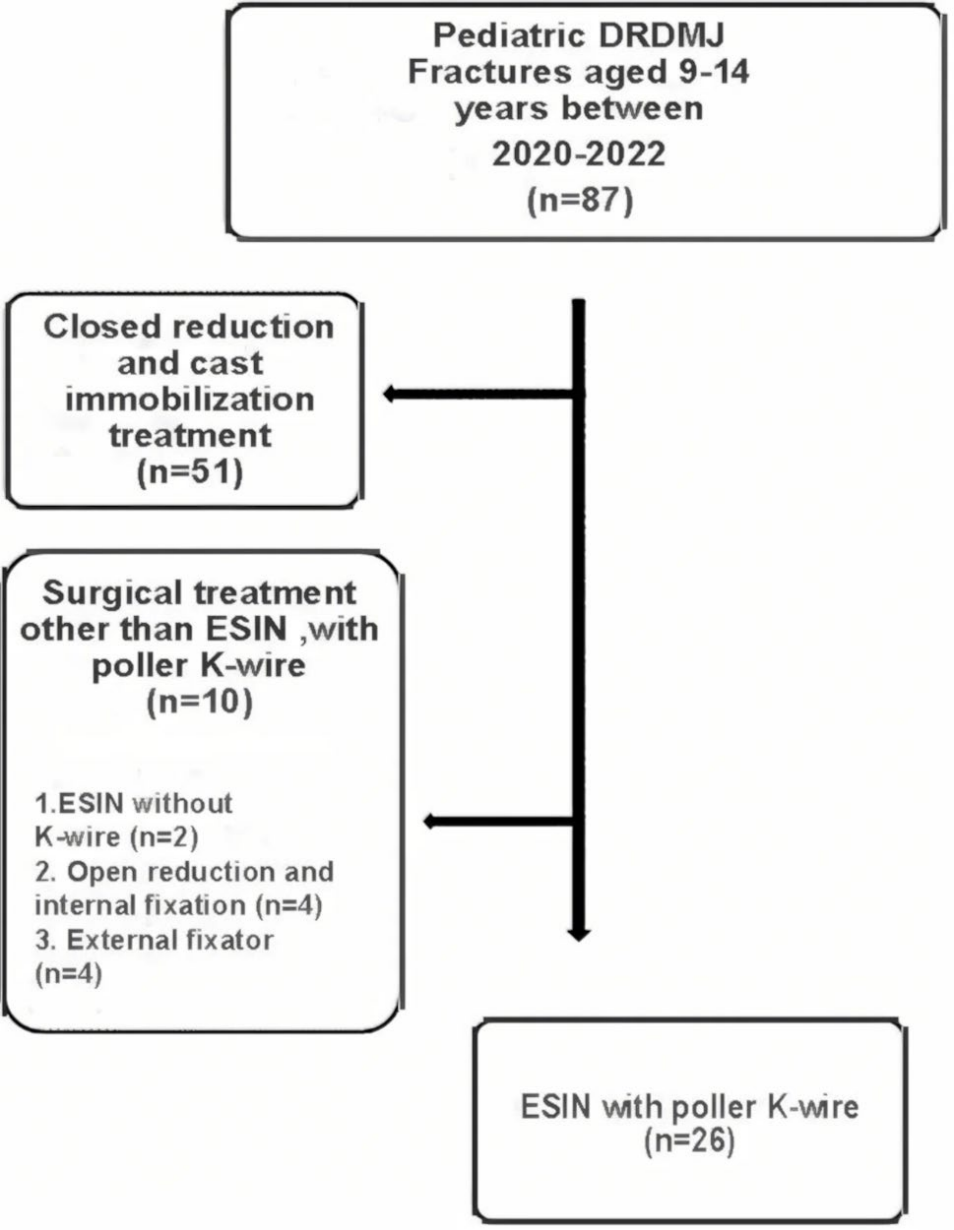
Flowchart of the patients who met inclusion criteria for the study

Pediatric DRDMJ fractures aged 9–14 years with limited remodeling capacity and incomplete skeletal maturity were included in the study. Between 2020 and 2022, 87 pediatric DRDMJ fractures were admitted to our clinic. In 51 patients, reduction parameters (Acceptable reduction parameters: <25° angulation in a lateral radiograph, < 10° angulation in an anteroposterior radiograph, translation ratio < 25% in lateral or anteroposterior radiograph) [13] were achieved with closed reduction and cast immobilization treatment at the first admission. Ten patients underwent surgical treatment other than ESIN with poller K-wire due to open fractures or comminuted fractures. All cases who underwent ESIN with a poller K-wire due to DRDM junctional fracture were included in the study (Fig. 1). The legal guardians of all participants were clearly informed about the procedure. Informed consent was obtained from the parents/guardians before surgery.

### Study Design and participants

**Fig. 2:**
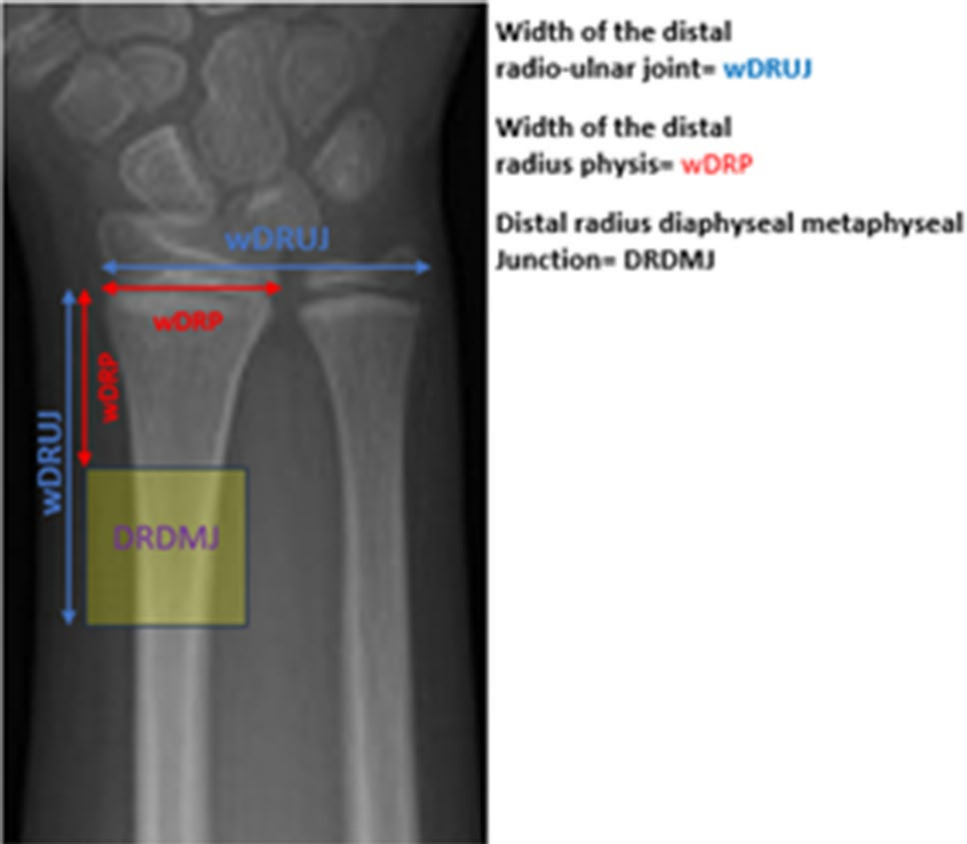
The distal radius diaphyseal metaphyseal junction is shown with a yellow square described by Lieber et al. [9]

Diaphyseal metaphyseal junction fractures previously described by Lieber et al. [9] were included in the study. While defining the DRDMJ, the width of the distal radius physis (wDRP) and the distal radio-ulnar joint (wDRUJ) were measured. The fractures between one wDRP and one wDRUJ distance from the physis were described as DRDMJ fractures (Fig. 2). In our study, we retrospectively evaluated patients who underwent ESIN with a poller K-wire in DRDMJ fractures between 2020 and 2022. All patients underwent surgery within two days. A single trauma-specialized orthopedic surgeon performed all surgeries under general anesthesia. The first emergency department admission radiographs obtained before reduction were not evaluated due to the instability of the fracture. Angular deformities and translation ratios in the coronal and sagittal planes were evaluated in the post-reduction, pre-operative radiographs of patients in whom adequate radiological parameters could not be achieved with closed reduction and plaster immobilization. Post-operative radiographs of all patients were evaluated to review the measurement of angulations in the frontal and sagittal planes and the translation ratio at the fracture site. On postoperative radiographs, sagittal and coronal angulations were defined as the angle formed between the long axis of the proximal fragment and a line oriented perpendicular to the epiphyseal plate [14]. In both anteroposterior (AP) and lateral radiographs, there should be no angle between the line-oriented perpendicular to the epiphyseal plate and the long axis of the proximal fragment. The angulations between these two lines were considered residual angulation. During growth, the limits of residual angulation that can be corrected by remodeling are < 10° in the coronal plane and < 30° in the sagittal plane [15]. Translation ratios were calculated by dividing the width of the non-contacting surface at the fracture line with the diameter of the fracture fragment on both AP and lateral radiographs (non-contact surface length of the two fragments/ diameter of the fracture fragment × 100%). There is a need for reintervention in cases where the translation at the fracture line is > 25% [16, 17]. Accompanying ulna fracture on preoperative radiographs was also evaluated. Coronal and sagittal plane angulations and translation rates at the fracture were recorded on the radiographs obtained immediately after the operation. Clinical follow-ups were performed in two weeks, four weeks, six weeks, three months, six months, and one year after surgery. Coronal and sagittal angulation and translation rates were evaluated in the radiographs obtained in the sixth week, as defined before. Fracture union and complications data were analyzed in clinical follow-ups. Poller K-wires were removed after fracture union was observed. Poller K-wires were removed after adequate union at four weeks. Plaster treatment was terminated at 4–6 weeks. ESINs were removed 3 to 6 months after the operation under general anesthesia.

At the final follow-up in the 12 months, the wrist’s range of motion and rotation of the forearm were assessed. The wrist joint function of the affected side was compared with that of the healthy side. The Price et al. grading system was used to evaluate the forearm functions of the patients at the last follow-up [18].

### Inclusion criteria

- Children aged 9–14 years,
- Failed closed reduction,
- No previous history of fracture,
- DRDMJ Fractures treated with a method ESIN with Poller K-wire.
- DRDMJ fractures with or without ulna fracture.

### Exclusion criteria

- Open or pathological fracture,
- Accompanying physis injury,
- Previous history of fracture at the forearm,
- Less than 12 months of follow-up.

### Examined variables

- Preoperative radiographic evaluation.
- Immediate postoperative radiographic evaluation.
- 6th week control radiographic evaluation.
- Last follow-up radiographic evaluation.

### Surgical technique

**Fig. 3:**
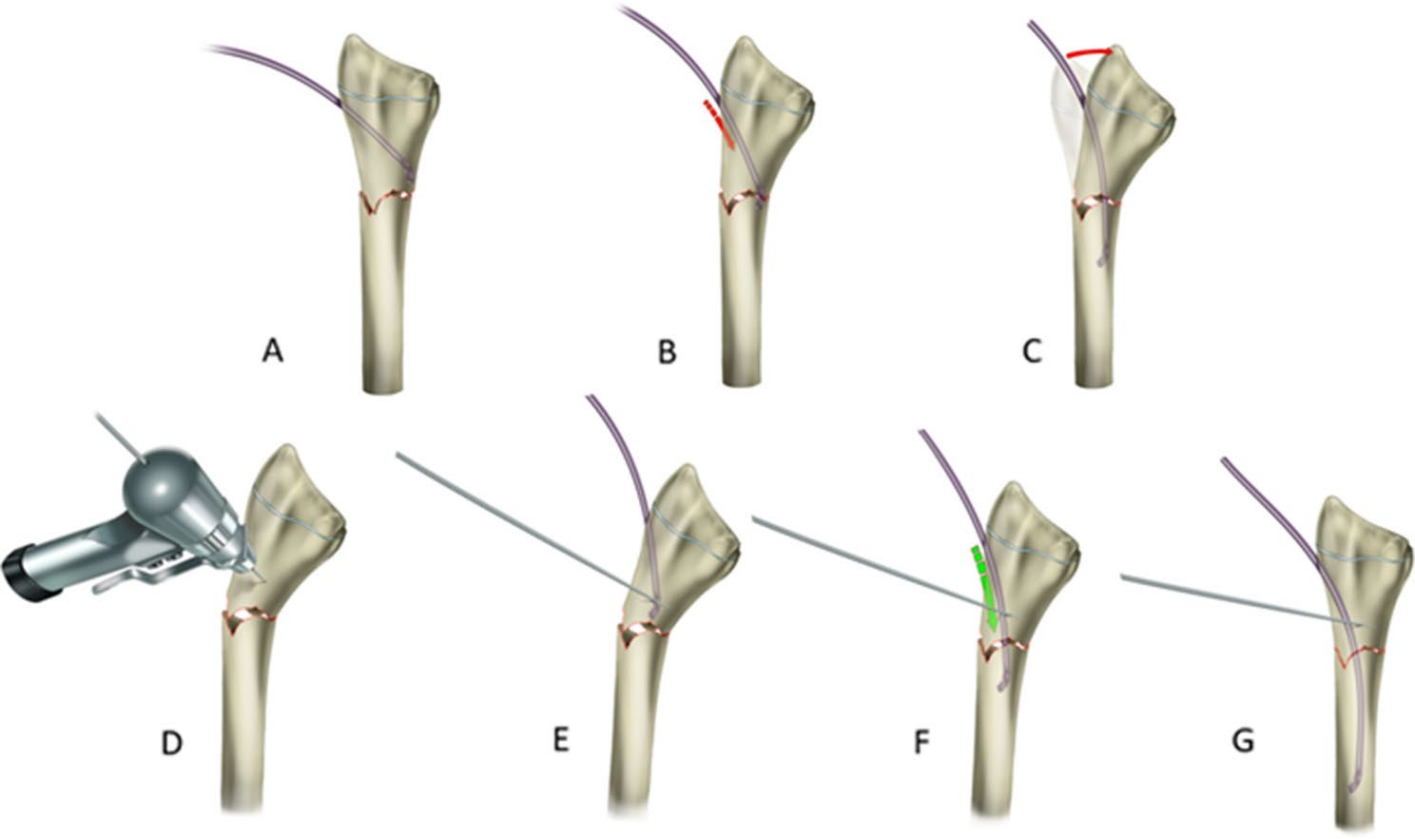
Illustration of ESIN application with poller K-wire in left DRDMJ fracture. (A, B, C) Loss of reduction due to ESIN application with classical technique in DRDMJ fractures. D: Poller K-wire application to the ulnar side of the distal fragment. E: Application of ESIN to the intramedullary space from the radial side of the poller K-wire, F: Advancing of the ESIN in the proximal fragment in the intramedullary space. G: Obtaining reduction with ESIN applied with Poller K-wire

The operation was performed under general anesthesia while the patient was supine, with the arm placed on the radiolucent side table. Each stage of the ESIN application was performed with the guidance of fluoroscopy. Before starting the application, ESINs of 2 or 2.5 mm were selected according to the diameter of the narrowest part of the radius medullary cavity with fluoroscopic control. In the first stage, closed reduction was performed under fluoroscopic guidance. In cases where the closed reduction was unsuccessful, a stab incision was made dorsal to the fracture line, and anatomical reduction was obtained with the help of curved Kelly forceps. After anatomical reduction, the entry point was determined under fluoroscopy guidance. Dorso-radial (through the Lister’s tubercle) and radial (slightly radial to Lister’s tubercle) entry points were used as entry points. The entry point is created around Lister’s tubercle, 0.5-1 cm proximal to the physis line. A 1.5 cm incision was made over the determined point. After subcutaneous soft tissue dissection, the curved Kelly hemostat forceps tip was opened, and the soft tissues were protected before applying the awl. The awl was placed at the determined point, and the location of the drill was confirmed by fluoroscopy. After creating the entry point, ESIN was applied in the intramedullary area with an oscillation maneuver. In case of loss of reduction due to ESIN application in fluoroscopic controls, the ESIN has withdrawn again with an oscillating maneuver. At the fracture level in the DRDMJ region, deformity with the apex on the radial side and radial translation of the distal fragment is observed due to ESIN application (Fig. 3). Retraction of the curved tip of the ESIN with oscillation creates the intramedullary area where the nail will settle within the fragment while reducing the fractured fragments while applying the nail after the poller K-wire.

**Fig. 4:**
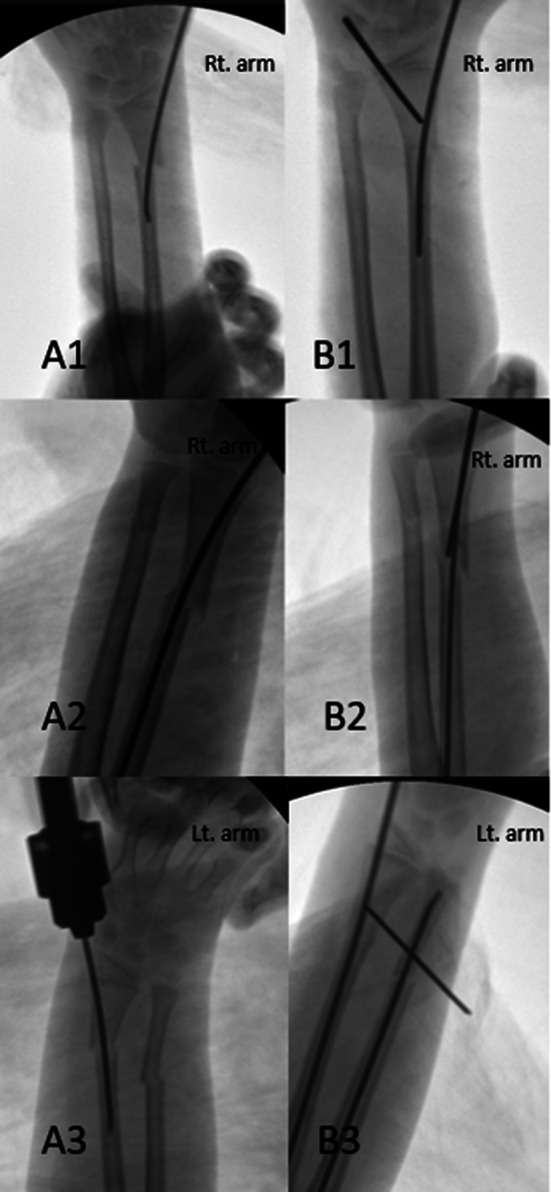
Correction of reduction loss due to ESIN application in the coronal plane with a poller K-wire at three patients. Loss of reduction due to ESIN application (A1, A2, A3). Correction deformity with poller K-wire (B1, B2, B3). Lt: left, Rt: right

**Fig. 5:**
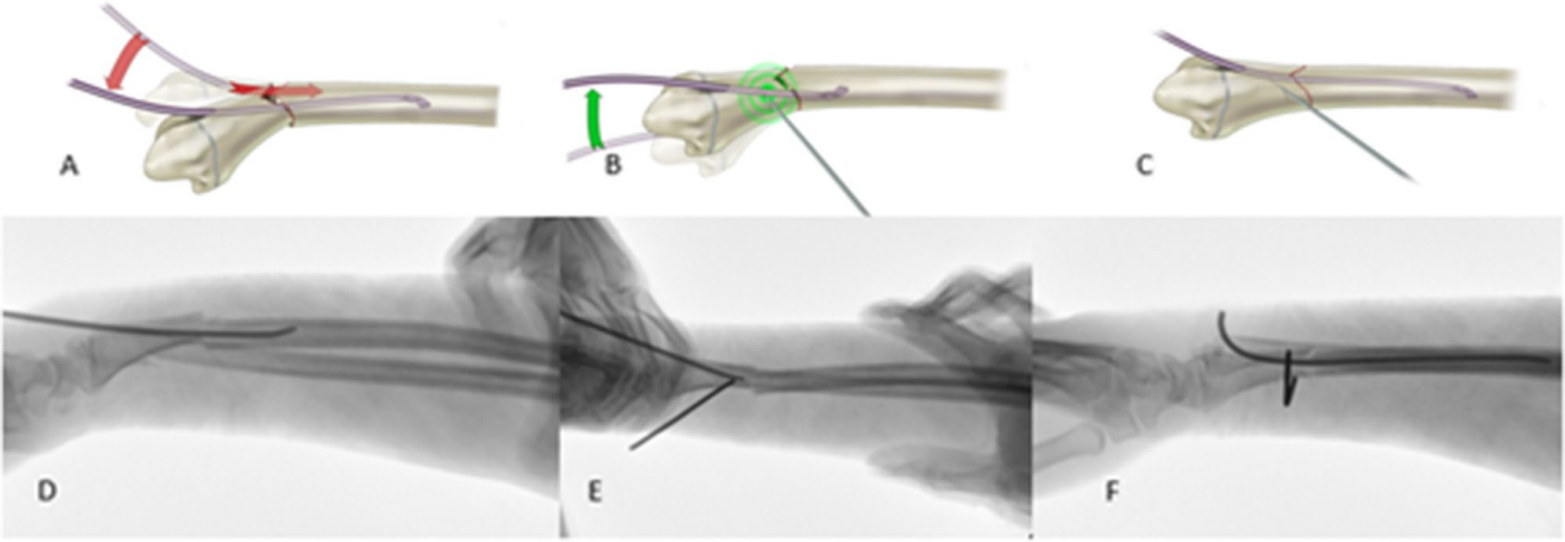
(A, B, C) Illustration of reduction loss due to ESIN application in sagittal plane and correction with poller K-wire. D: Sagittal deformity development due to ESIN application. E: Volar poller K-wire application with ESIN. F: Correction of loss of reduction in the sagittal plane with minimal angulation after Poller K-wire application

Before re-applicating ESIN, the poller K-wire was applied to the distal fragment on the concave side of the displacement, where we wanted the fractured fragments to move under fluoroscopic control. It was applied to the distal fragment with a large metaphyseal area because it allowed adequate correction and nail passage. A 0.5 cm skin incision was made for the poller K-wire application. During the application of poller K-wire, tendon and soft tissue structures were protected by using a sleeve. 1.6 mm K-wires were preferred for the poller K-wire. In the first application performed without a poller K-wire, the point where the ESIN passed closest to the fracture line in the distal fragment was determined, and a poller K-wire was applied to this point, ensuring ulnar translation of the distal fragment and correction of the angular deformity. After the application of the Poller K-wire, ESIN was passed through the radial side of the Poller K-wire under fluoroscopy control. The poller K-wire creates a lever effect on the distal fragment with the contact of the ESIN, enabling the translation of the distal fragment to the ulnar side and correcting the angulation (Fig. 4). The reduction was confirmed after ESIN passed through the proximal fragment in the intramedullary area under fluoroscopic guidance. In cases where the reduction was insufficient, the poller k wire was applied more radially to increase the fragment movement and angular correction during ESIN passage in the proximal fragment. ESIN was reapplied with a poller K-wire to the volar aspect of the distal fragment for the angulation in the dorsal apex that occurred in the sagittal plane due to the ESIN application (Fig. 5). After the application, reduction and stability were checked with fluoroscopy. After the radius fixation, the distal ulna fracture reduction was checked under C-arm fluoroscopy. If needed, antegrade ESIN was applied for ulna fracture. The tail of ESIN was then cut off and located under the subcutaneous tissue.

### Postoperative care

All patients were immobilized in a short-arm cast for four to six postoperative weeks to prevent poller K-wire migration. If sufficient union was observed, the Poller K-wire was removed at four weeks, and the short-arm cast treatment was terminated at 4 to 6 weeks. The free and full-arm motion was allowed after that.

### Primer & seconder outcomes

Coronal and Sagittal plane translation.

Coronal and Sagittal plane angulation.

Coronal and sagittal plane angulation change at the 6-week follow-up.

Coronal and sagittal plane translation change at the 6-week follow-up.

Forearm and wrist range of motion.

Forearm and wrist functional scores according to Price et al. grading system.

### Ethics

The approval of the Ethics Committee has been obtained from the institution to which the researchers are affiliated (2023-14/93). Participation in the study has been voluntary and conducted following the principles of voluntarism. Informed consent has been obtained from all participants’ legal guardians.

### Statistical analysis

Statistical analysis was performed using Statistical Package for Social Sciences version 29.0 software in the statistical analysis of the study for Windows (IBM SPSS Statistics for Windows, Version 29.0. Armonk, NY: IBM Corp., USA). Descriptive statistics are numbers, percentages, mean, standard deviation, median, minimum, and maximum values. Several observations of categorical variables were given as n. The normality of the variables was tested with Kolmogorov-Smirnov and Shapiro-Wilk tests. Wilcoxon’s Signed Rank test was used to compare the range of motion of the injured side compared to the uninjured side. Changes in radiological parameters were analyzed in 3 different periods (preoperatively, postoperatively and 6th postoperative week) with the repeated measures ANOVA method. Before the repeated measures ANOVA test, Mauchly’s Test of Sphericity assumption was tested. Since Mauchly’s Test of Sphericity assumption was not met, the Greenhouse-Geisser correction was made. Differences between the times (Preoperative, immediate postoperative, and postoperative 6th week) where significant differences were detected as a result of Repeated Measures ANOVA were tested using the Bonferroni multiple comparison test. For all statistical analyses, *p* < 0.05 was considered statistically significant.

## Result

All cases who underwent ESIN with a poller K-wire due to DRDMJ fracture were included in the study. Data from seventeen male and nine female patients with an average age of 10.9 ± 1.45were analyzed (Table 1).

**Table 1:** Demographic and clinical features of patient’s

Patient demographics (*n* = 26)	
Age (years; mean ± sd)	10.9 ± 1.45
Sex (Female/male )	9/17
Side (left/right)	6/20
Associated ulnar fracture	11
ESIN for ulna fracture	7
Duration of surgery (min; mean ± sd)	44 ± 9
Removal of the poller K-wire (week)	4
Removal of the short arm cast(week)	4.5 ± 0,9
Removal of the ESIN (month; mean,min-max)	4.2 [3–6]
Follow-up (month; mean, min-max)	13.6 [12–14]

ESIN: Elastic stable intramedullary nail, SD: Standard deviation, Min: minute, Min-max: Minimum-maximum

Eleven of the patients had concomitant ulna fractures. ESIN was applied to seven patients when displacement or instability was detected in the ulna fracture after radius fixation in fluoroscopic controls. After radius fixation in five patients, the reduction was achieved in the ulna, and no instability was detected, so no additional fixation was applied. The mean operation time was 44 ± 9 min. The anatomical reduction was achieved in all cases accompanied by ulna fracture, and no reduction loss for ulna fracture was observed in clinical follow-ups. No problem, such as nonunion, was encountered for the ulna fracture. The mean angulations in the coronal and sagittal planes measured on preoperative radiographs were 18.0 ± 11.06° and 35.0 ± 12.49°, respectively. The mean coronal and sagittal plane translation ratios measured on preoperative radiographs were 30.0 ± 9.78% and 45.0 ± 15.49%, respectively. The mean coronal plane angulation in the radiographs taken immediately after the surgery of the patients were 4.0 ± 1.62°, and the sagittal plane angulations were 3.0 ± 1.26°. On immediate post-operative radiographs, the mean translation rates in the coronal and sagittal planes were 6.0 ± 1.98% and 5.0 ± 2.02%, respectively. No change was observed in the translation ratios in both planes in the radiographs obtained at the 6th-week follow-up. The mean angulation in the coronal and sagittal planes measured on 6th-week radiographs was 4.0 ± 1.72°and 3.0 ± 1.16°, respectively. (Table 2).

**Table 2:** Radiographic evaluation data of pre-operative, immediately after surgery, and six weeks after fracture healing postoperatively

Variables	Pre-operative (Mean ± SD)	Immediate post-operative (Mean ± SD)	Six weeks (Mean ± SD)	P value
Coronal plane translation ratio (%)	30.0 ± 9.78	6.0 ± 1.98	6.0 ± 1.98	< 0.001^a,b^ > 0.05 ^c^
Sagittal plane translation ratio (%)	45.0 ± 15.49	5.0 ± 2.02	5.0 ± 2.02	< 0.001^a,b^ > 0.05^c^
Coronal plane angulation (°)	18.0 ± 11.06	4.0 ± 1.62	4.0 ± 1.72	< 0.001^a,b^ > 0.05^c^
Sagittal plane angulation (°)	35.0 ± 12.49	3.0 ± 1.26	3.0 ± 1.16	< 0.001^a,b^ > 0.05^c^

SD = Standard Deviation,

^a^change from preoperative to immediate postoperative,

^b^change from preoperative to immediate postoperative,

^c^change from immediate post-operative to six weeks after surgery

Significant differences were observed in all pre-operative and immediate post-operative radiological parameters (*p* < 0.001). There was no statistically significant difference between the values ​​obtained from the immediate postoperative and 6th-week follow-up radiographs (*p* > 0.05). Union was observed in all patients 4th- and 6th-week control radiographs. One of the patients had skin irritation and superficial infection due to radius ESIN application and was followed up with dressing and antibiotic therapy. No tendon irritation, rupture, or neurovascular damage was observed in any of the patients. The forearm and wrist range of motion data obtained at the last follow-up of the patients are presented in Table 3.

**Table 3:** Clinical outcomes at the last follow-up

Range of motion	Injured side, degree Median (min-max)	Uninjured side, degree Median (min-max)	P value*
Flexion	84.00(82–85)	84.00(83–85)	0.42
Extension	79.00(78–80)	79.00(79–80)	0.25
Supination	84.00(82–85)	84.00(83–85)	0.55
Pronation	84.00(82–85)	84.00(83–85)	0.36

* Wilcoxon’s Signed Rank test (*P* < 0.05 was identified as statistically significant)

**Fig. 6:**
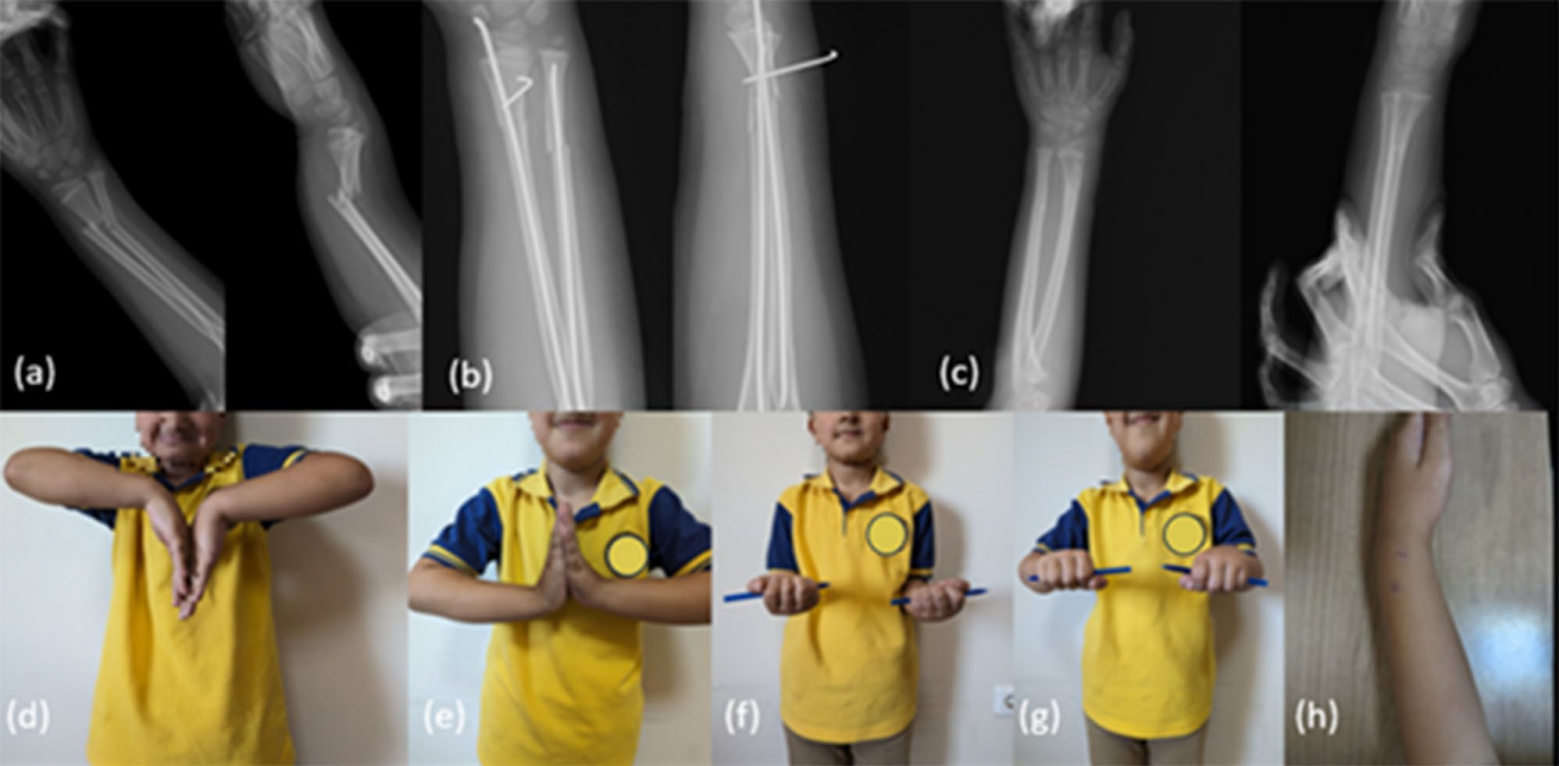
A male patient, 11 years old, with a left DRDMJ fracture accompanied by an ulna fracture. Anterior-posterior and lateral radiographs: (a) preoperative; (b) day one postoperative; (c) 3 months postoperative. Wrist and forearm range of motion examination at last follow-up: (d) flexion; (e) extension; (f) supination; (g) pronation; (h) scar

In terms of forearm and wrist movements, when compared to the healthy side, no significant difference was observed in terms of flexion, extension, pronation, and supination(*p* > 0.05). At the last follow-up, none of the patients had a forearm rotation loss of more than 15 degrees and no limitations in their strenuous activities (Fig. 6). The patients recovered with an excellent score according to the grading system of Price et al.

## Discussion

As a result of our study, 1 and 2 degrees of changes were observed in the coronal and sagittal planes, respectively, in the 6th-week follow-up of the patients due to the application of TEN with the poler K-wire. All patients healed with fracture angulation of less than 10 degrees at the 6th week follow-up. In the 6th week follow-up of the patients, no change was observed in the fracture apposition rates in both the sagittal and coronal planes. As a result of the study of Tarr et al. [19], they observed a 13% loss of forearm rotation in patients who recovered with more than 10 degrees of angulation. In their study, Sato et al. [8] considered angulation more significant than 10 degrees after surgery as a malunion that affects clinical results. In our research, angulation of more than 10 degrees was not observed in any cases in which ESIN was applied with the poller K-wire, and the forearm rotation limitation of the patients improved below 10%.

The fracture healing capacity is low in this region because the fracture location is far from the metaphyseal area with good blood supply, and the plasticity is low [20–22]. Satisfactory alignment is not achieved due to the displacing effect of the muscles and tendons passing close to the fracture side [23]. Surgical treatment is recommended for fractures in which closed reduction fails due to high complication rates due to reduction losses and malunions [24–26]. Closed reduction and cast immobilization treatment mostly give successful results in patients under the age of nine with higher remodeling capacity and a more comprehensive range of acceptable reduction parameters [27, 28]. There is no consensus on surgical treatment for patients with irreducible DRDMJ fractures over the age of 9 years. Lieber et al. defined a fracture application that remains too distal for K-wire application and too proximal for ESIN application for fractures in the DRDJM region [9]. Reduction losses were observed in the follow-ups due to insufficient K-wire fixation of fractures due to applications with very high angles to the fracture [8]. Transepiphyseal intramedullary applications have been described previously due to the difficulty of applying ESIN and the inability to obtain proper alignment [4, 9, 29]. In trans-physeal applications, there is a risk of injury to the growth plate due to multiple entry attempts [30, 31].

In our practice, ESIN is applied 1.5-2 cm proximal to the physeal plate by the classical definition, and poller K-wires are applied close to the fracture line. There are publications on changing the entry point to achieve more successful results in ESIN applications [32, 33]. In our applications, even if the entry point was taken posteromedially, it was observed that sagittal plane deformity developed due to the transition region of the DRDMJ and the narrow medullary cavity. After ESIN application with poller K-wire, alignment was provided in the sagittal plane (Fig. 3). Other techniques described in DRDMJ fractures are the previously bent ESIN and short double ESIN applications [6, 7, 11]. The narrow medullary cavity is also disadvantageous in these techniques regarding iatrogenic fracture. No iatrogenic fracture was observed in the technique we described. Antegrade ESIN applications have been described in DRDMJ fractures [34]. Due to limited literature data on antegrade ESIN application, an apparent rate regarding the risk of posterior interosseous nerve injury cannot be revealed. No safe zone in proximal radius interventions has been defined for the pediatric age group. In antegrade ESIN applications, it is necessary to consider the anatomical variations of the posterior interosseous nerve [35]. The poller screw application is widely used to treat metaphyseal fractures during intramedullary nailing [36]. Poller screw applications both provide reduction and increase fixation strength during intramedullary nail application in metaphyseal fractures [37, 38]. After ESIN application with poller K-wire, the intraoperative reduction was improved, and sufficient fixation strength was provided in clinical follow-ups. ESIN application with a poller K-wire is a minimally invasive technique, and no delayed union or neurovascular damage was observed due to the application. OR-IF is recommended for older children because it provides more stable fixation and satisfactory reduction [12]. However, some complications could be seen, such as more extensive skin scars, slower healing, a high rate of refractures, and growth plate injury [39, 40]. Some publications also show that OR-IF and intramedullary fixation give similar results regarding significant complication rates [41]. No delayed union or nonunion was observed in cases where ESIN was applied with the poller K-wire. None of the patients required additional surgical intervention.

Many modified ESIN methods for DRDMJ fractures have been described in the literature. As a result of antegrade nail applications, postoperative angulation of less than 5° in both planes and translation of less than 25% in the coronal plane and less than 5% in the sagittal plane were observed [34]. As a result of short double ESIN application for DRDMJ fractures, anatomical reduction was achieved in 20 patients, and acceptable and good results were observed in the remaining patients [11]. As a result of posteromedial ESIN application, it was observed that there was a 7° (range 3°-18°) residual deformity in the sagittal plane and a 6.5° (range 4°-12°) residual deformity in the coronal plane. No information was provided regarding translation rates [33]. As a result of Epinbolic K-wire application, an average of 6.6 ± 4.5 degrees of angulation was observed in the sagittal plane and 2.4 ± 3.4 degrees in the coronal plane. No data are given regarding translation amounts [42]. As a result of pre-bending ESIN applications, 7% and 5% translation were observed in the coronal and sagittal planes, respectively, and 5° and 7° angulation were observed in the sagittal and coronal planes, respectively [7]. As a result of transepiphyseal K-wire fixation, 2.25° (range 0°-8.8°) and 4.61°(range 0°-15.8°) degrees of angulation were observed in the coronal and sagittal planes, respectively, and 16.44% and 14.89% translation were observed in the coronal and sagittal planes, respectively [29]. In our study, as a result of ESIN application with poller K-wire, 4.0 ± 1.62° and 3.0 ± 1.26° angulations and 6.0 ± 1.98% and 5.0 ± 2.02% translation were observed in the coronal and sagittal planes, respectively.

The main limitation of our study is that a small population was evaluated retrospectively. The second limitation of our study is that the incidence of DRDMJ fractures was low, and the treatment modality was not standardized, so a control group could not be established. The third limitation of our study is that DRDMJ fractures accompanied by ulna fractures cannot be evaluated separately due to the low number of cases. The last limitation is the long learning curve for precise and accurate poller K-wire placement. In both coronal and sagittal plane deformities due to ESIN application, the apex of the deformity should be evaluated very carefully under fluoroscopy control, whether there is not enough intramedullary space for ESIN after both volar and radial poller K-wire application.

## Conclusion

ESIN application with poller K-wire is an effective, safe, minimally invasive method for the surgical treatment of pediatric DRDMJ fractures. Poller K-wire application is practical in providing reduction during surgery and increasing stability in clinical follow-ups.

## Data Availability

The datasets used and/or analyzed during the current study are available from the corresponding author on reasonable request.
