# Correction: Stable elastic nail application with poller K-wire for Irreducible distal radius metaphyseal-diaphyseal Junction fractures in preadolescents: a new operative technique

**DOI:** 10.1186/s12891-025-08517-y

**Published:** 2025-03-18

**Authors:** Levent Horoz, Mehmet Fevzi Cakmak, Cihan Kircil

**Affiliations:** Faculty of Medicine Orthopedics and Traumatology Clinic, Kırşehir Ahi Evran University, Kirsehir, Turkey

**Correction** **:** **BMC Musculoskelet Disord 25, 228 (2024)**

**https://doi.org/10.1186/s12891-024-07358-5**

Following the publication of the original article [1], In the Patients and methods section of the article, under the subheading “Study population” the date range in the sentence "Between 2019 and 2022, 87 pediatric DRDMJ fractures were admitted to our clinic." was given incorrectly. The correct sentence should be "Between 2020 and 2022, 87 pediatric DRDMJ fractures were admitted to our clinic."

Moreover, the date in the flow chart of the patients in Fig. 1 was given incorrectly as 2019 and 2022 and should have been “2020-2022”. The flow chart with the correct date has been included below.

**Fig. 1:**
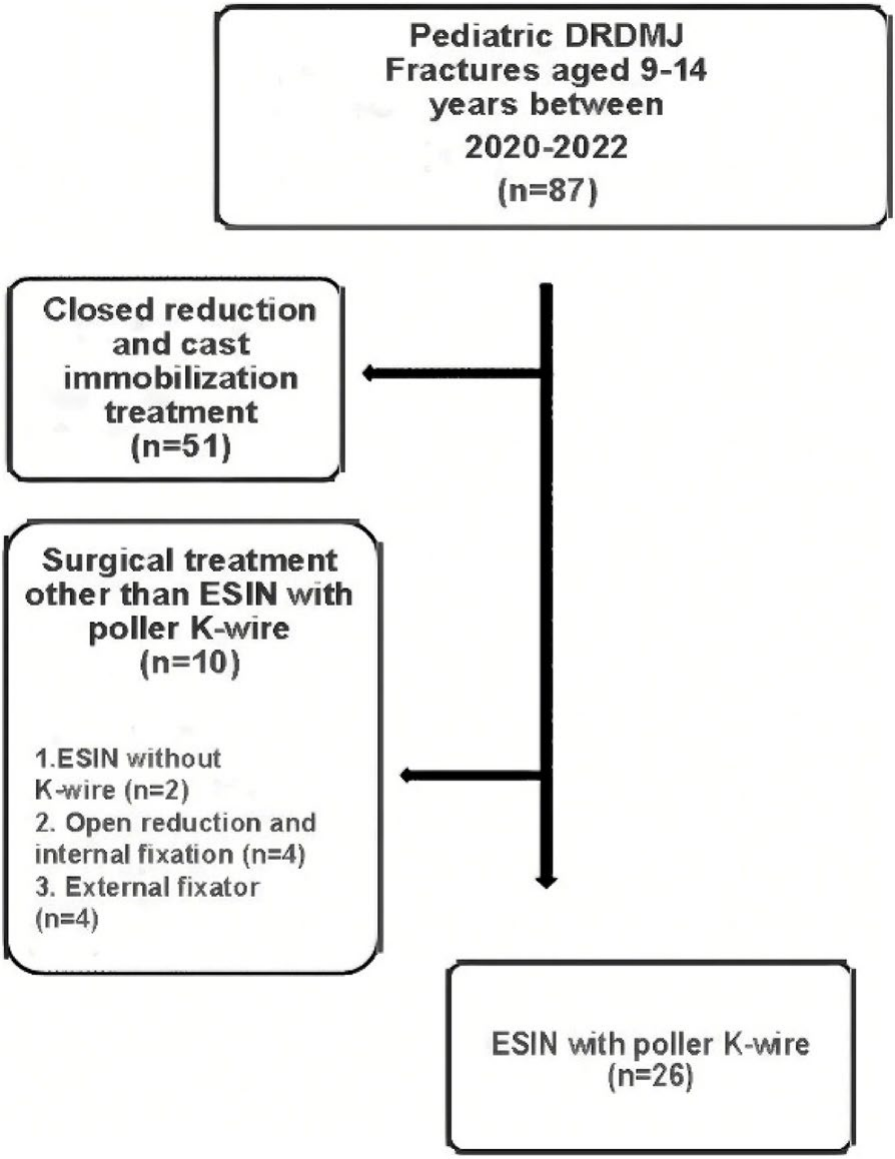
Flowchart of the patients who met inclusion criteria for the study

The original article [1] has been updated.
